# Health and Labor Market Outcomes Among Older Adults: Causal Evidence From Korean Panel Data and Dynamic Modeling

**DOI:** 10.1093/geroni/igaa057.1489

**Published:** 2020-12-16

**Authors:** Hoolda Kim, Sophie Mitra

**Affiliations:** 1 Black Hills State University, Spearfish, South Dakota, United States; 2 Fordham University, Bronx, New York, United States

## Abstract

Health and work are two key determinants of economic insecurity and wellbeing. Since economic insecurity may rise with age due to the deterioration of health and work ability, it is essential to study the causal relationship between health and labor market outcomes among older people. In Korea, the size of the older population has grown rapidly in recent decades but the pension system remains limited in terms of its reach and generosity, leaving a considerable number of older people suffering from economic insecurity. To investigate the two-way causal links between health and labor market outcomes, we use unique data on middle-aged and older Koreans from 12 waves of the Korea Welfare Panel Study (2006-2017) and two dynamic modeling approaches: the Arellano-Bond Generalized Method of Moment Model (AB-GMM) and the Maximum Likelihood Structural Equation Model (ML-SEM). Results point at gender differences regarding the direction of the causal link between health and labor market outcomes. Men are more likely to be in good health due to higher income or paid work. Women are more likely to earn a higher income and to work as paid employees because of good health. These effects particularly hold for Koreans aged 55-74 in urban areas.

